# Propelling DNA Computing with Materials’ Power: Recent Advancements in Innovative DNA Logic Computing Systems and Smart Bio‐Applications

**DOI:** 10.1002/advs.202001766

**Published:** 2020-11-09

**Authors:** Daoqing Fan, Juan Wang, Erkang Wang, Shaojun Dong

**Affiliations:** ^1^ State Key Laboratory of Electroanalytical Chemistry Changchun Institute of Applied Chemistry Chinese Academy of Sciences Changchun Jilin 130022 China; ^2^ University of Science and Technology of China Hefei Anhui 230026 China; ^3^ Present address: Institute of Chemistry The Hebrew University of Jerusalem Jerusalem 91904 Israel

**Keywords:** biomaterials, DNA logic computing, functional nucleic acids, logic‐directed smart bio‐applications, nanomaterials

## Abstract

DNA computing is recognized as one of the most outstanding candidates of next‐generation molecular computers that perform Boolean logic using DNAs as basic elements. Benefiting from DNAs’ inherent merits of low‐cost, easy‐synthesis, excellent biocompatibility, and high programmability, DNA computing has evoked substantial interests and gained burgeoning advancements in recent decades, and also exhibited amazing magic in smart bio‐applications. In this review, recent achievements of DNA logic computing systems using multifarious materials as building blocks are summarized. Initially, the operating principles and functions of different logic devices (common logic gates, advanced arithmetic and non‐arithmetic logic devices, versatile logic library, etc.) are elaborated. Afterward, state‐of‐the‐art DNA computing systems based on diverse “toolbox” materials, including typical functional DNA motifs (aptamer, metal‐ion dependent DNAzyme, G‐quadruplex, i‐motif, triplex, etc.), DNA tool‐enzymes, non‐DNA biomaterials (natural enzyme, protein, antibody), nanomaterials (AuNPs, magnetic beads, graphene oxide, polydopamine nanoparticles, carbon nanotubes, DNA‐templated nanoclusters, upconversion nanoparticles, quantum dots, etc.) or polymers, 2D/3D DNA nanostructures (circular/interlocked DNA, DNA tetrahedron/polyhedron, DNA origami, etc.) are reviewed. The smart bio‐applications of DNA computing to the fields of intelligent analysis/diagnosis, cell imaging/therapy, amongst others, are further outlined. More importantly, current “Achilles’ heels” and challenges are discussed, and future promising directions of this field are also recommended.

## Graphical abstract

As one of the most fascinating candidates of next‐generation molecular computers, DNA computing has attracted substantial interests, and achieved burgeoning developments in recent decades. In this review, recent achievements of innovative DNA logic computing systems based on various “building‐block” materials and corresponding smart bio‐applications are summarized. Furthermore, current “Achilles’ heels” and future promising directions are discussed.

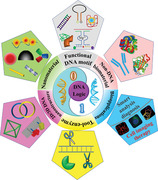

## 1 Introduction

In the current information technology (IT) age, silicon‐templated semiconductor computers play the predominant roles and are confirmed as essential tools for communicating, working, and even scientific research.^[^ ^1^, ^2^ ^]^ However, the word “computation” was proposed much earlier than the appearance of transistor circuits.^[^ ^3^, ^4^ ^]^ Since George Boole used mathematical numbers to perform logic operations that assign “false” value to “0” and “true” value to “1” in the form of binary bits,^[^ ^3^ ^]^ the concept of Boolean logic (basic‐principle of silicon computers) was built. Logic gates are mineralized devices that implement Boolean logic, and they are stimulated by binary‐encoded inputs (0 or 1) and can generate corresponding binary outputs (0 or 1).^[^ ^5^, ^6^, ^7^ ^]^ For modern semiconductor IT system which acts as one approach of Boolean logic, electronic triggers (voltage or current) are employed as inputs and outputs. However, according to the quantum physics theory prediction, the miniaturization technology will hit its physical limit before long.^[^ ^8^ ^]^ Under this background, molecular engineers aim at searching for novel and promising substitutes for logic computing to surmount above limitations, which thereby conduces to the emergence of “molecular logic.” As molecular‐level functional mimics of silicon logic counterparts, “molecular logic” has attracted broad interests and paved unique horizons for implementing diverse smart applications.^[^ ^9^ ^]^

**Figure 1:**
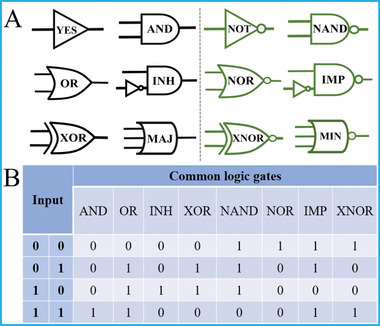
A) Equivalent logic circuit of basic logic gates and B) corresponding truth tables of 2‐input ones.

For molecular logic gates, the binary‐encoded molecule or light^[^ ^10^, ^34^ ^]^ is applied as input stimuli (absence, 0; presence, 1), and small molecules, optical (colorimetric, luminescent), or electrochemical signals are taken as outputs (high, 1; low, 0). And each kind of logic gate has a special input–output correlation prototype that follows the truth table (**Figure** **1**) formulated by George Boole's characteristic perspectives.^[^ ^11^ ^]^ Inspired by the seminal molecular AND logic gate that created by Prof. de Silva in 1993,^[^ ^12^ ^]^ this field has sprung up like mushrooms and varieties of logic devices using distinctive building components (organic molecules, nucleic acids, enzymes etc.) are fabricated.^[^ ^13^, ^14^, ^15^ ^]^ From our point of view, molecular logic devices can be classified into common logic gates, advanced logic devices for specific arithmetic functions or non‐arithmetic information‐processing, other general cascaded/concatenated logic circuits, versatile logic library, non‐Boolean logic, and so on, see **Table** **1**. The detailed operating principle of these logic devices will be elaborated in Section 2, accompanied with representative examples. It should be noted that there are many different kinds of logic devices and this general classification is made on the basis of our experience and unique insights into this field, which is beneficial to demonstrate this comprehensive review more methodically. Considering that molecular logic is molecular‐level functional analogue of semiconductor transistors, much more logic devices can theoretically be achieved and the devices illustrated herein is just the tip of an iceberg.

**Table 1:** Categories of various logic devices

Logic devices	Logic contents or functions	
Common logic gates	YES, AND, OR, INH, XOR, MAJORITY (MAJ), NOT, NAND, NOR, IMP, XNOR, MINORITY (MIN), and others.	
Advanced logic devices for specific arithmetic functions.	Half adder (HA)	XOR (SUM)+AND (CARRY)
	Half subtractor (HS)	XOR(DIFFERENCE)+INH (BORROW)
	Full adder (FA)	Integration of HA and OR
	Full subtractor (FS)	Integration of HS and OR
	Others	‐
Advanced logic devices for non‐arithmetic information‐processing.	Encoder/decoder	Mutual conversion of binary data/code
	Multiplexer/demultiplexer	Compression/decompression of binary data
	Parity generator/checker (pG/pC)	Detect error in data transmission (cascades of XOR)
	Voter	MAJ gate
	Keypad lock	Sequential logic
	Parity‐checker (Pc)	Distinguish even/odd natural numbers
	Others	‐
Other general cascaded/concatenated circuits	Functional integration of varieties of logic gates	
Versatile logic library	Multifarious logic gates/devices based on a universal operating system.	
Non‐Boolean logic	Multi‐valued logic or Fuzzy logic	
Other ones	‐	

As one charming alternative of molecular computing, DNA computing has evoked substantial interests and gained rapid developments recently. With the prosperous advancements of DNA nanotechnology that pioneered by Ned Seeman,^[^ ^16^, ^17^, ^18^ ^]^ DNA is not considered as one kind of pure genetic‐code carriers, but has emerged as exquisite engineering materials for logic computing. Since Adleman's ground breaking work that applied DNA as basic unit to solve Hamilton path problems,^[^ ^19^ ^]^ multifarious DNA logic systems were engineered successfully. Among which, the binary‐encoded DNAs or other triggers are used as inputs (absence “0,” presence “1”), and DNA strands, optical (fluorescent, visual, chemiluminescent) or electrochemical signals are outputs (low “0,” high “1”). The inherent superior merits of DNA, such as easy synthesis, low cost, great flexibility, precise Watson–Crick principle, programmed nanostructures, and powerful computing parallelism, have made DNA computing the most ingenious candidate of molecular computing. Moreover, the combination of Boolean logic's stringency with DNAs’ excellent biocompatibility also stimulated smartly designed bio‐applications of DNA computing to bioanalysis/diagnostics, bioimaging, drug load/release, and so on.^[^ ^8^, ^9^, ^16^ ^]^

In this review, we first systematically classify diverse logic devices into different categories (from common logic gates to versatile logic library and so on, See Table 1), and then illustrate the operating principles and functions of these logic devices. After that, emphasis is put on state‐of‐the‐art DNA computing systems using varieties of materials as building components, including representative functional DNA motifs (aptamer, metal‐ion dependent DNAzyme/DNA nanostructure, G‐quadruplex, i‐motif, triplex, molecular beacon (MB), etc.), DNA tool‐enzymes (nicking enzyme, polymerase, exonuclease), non‐DNA biomaterials (natural enzyme, protein, antibody), nanomaterials (AuNPs, magnetic beads, graphene oxide, polydopamine nanoparticles, carbon nanotubes, DNA‐templated nanoclusters, upconversion nanoparticles, quantum dots) or polymers, 2D/3D DNA nanostructures (circular/interlocked DNA, DNA tetrahedron or polyhedron, DNA origami). We also highlight inspiring bio‐applications of DNA computing to the areas of intelligent bioanalysis and diagnosis, cell imaging/therapy, and so on. More importantly, we discuss current obstacles and challenges, and also suggest future perspectives of this field. We hope that this review will not only benefit the newcomers in the field of DNA computing, but also enlighten future progress of this highly active area assisted with DNA nanotechnology and advanced functional materials.

## 2 Functions and Principles of Miscellaneous Logic Devices

Before introducing DNA computing systems, we initially make a general classification for multifarious logic devices (see Table 1), and discuss corresponding logic principles and functions as follows in this section. The logic devices are classified into following categories: common logic gates, advanced logic devices for specific arithmetic functions or non‐arithmetic information‐processing, other general cascaded/concatenated logic circuits, versatile logic library, non‐Boolean logic and others. It should be noted that this classification is made according to our unique insights into this area, which is beneficial to illustrate the functions of different logic devices more reasonably.

### 2.1 Common Logic Gates

The elementary logic gates are the foundation of molecular computing and also fundamental components for varieties of sophisticated logic devices. Typically, the common logic gates include YES, AND, OR, INHIBIT, XOR, MAJORITY gates, and the ones with contrary functions (NOT, NAND, NOR, IMPLICATION, XNOR, and MINORITY) and others. Their equivalent logic circuits and truth tables are shown in Figure 1 and corresponding operating principles are illustrated below.

For the simplest 1‐input YES gate, the absence of input (0) will generate output (0), whereas the presence of input (1) will generate output (1), following the “0 to 0” and “1 to 1” mapping mode. For AND gate, only the concomitant presence of all inputs (11) could produce output (1), otherwise the output will be (0). For OR gate, the presence of any input could generate output (1) and only the absence of all inputs (00) yields output (0). For INHIBIT gate, one of the inputs acts as the inhibitory element, only in the absence of this element and co‐existence of all the other inputs could generate output (1). For the XOR gate which is usually hard to realize at molecular‐level, it has unique input‐to‐output pattern. The presence of any input (01 or 10) will produce output (1), while the concomitant absence or presence of all inputs (00 or 11) yields output (0). For the MAJORITY gate that can be used in fault‐tolerant computing, its output will be TRUE value (1) only in the presence of more than half inputs, otherwise the output is FALSE (0). All the above demonstrates the detailed principles of 6 common logic gates, and the input–output correlations of NOT, NAND, NOR, IMP, XNOR, and MINORITY gates are just opposite to these 6 ones described above, respectively. These elementary logic constituents play crucial roles in diverse multifunctional logic devices.

### 2.2 Advanced Logic Devices for Specific Arithmetic Functions

**Figure 2:**
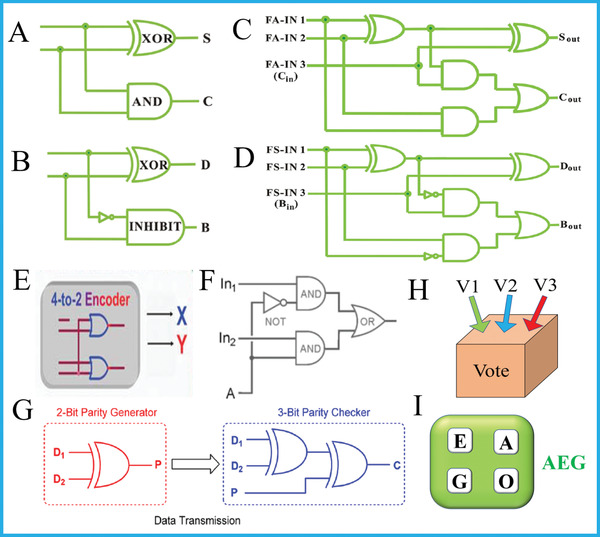
Equivalent logic circuit of A) HA, B) HS, C) FA, and D) FS. Reproduced with permission.^[^ ^69^ ^]^ Copyright 2015, Wiley‐VCH; E) The 4‐to‐2 encoder, Reproduced with permission.^[^ ^26^ ^]^ Copyright 2012, Springer Nature; F) 2‐1 multiplexer. Reproduced with permission.^[^ ^29^ ^]^ Copyright 2008, Wiley‐VCH; G) The pG/pC system for error detection in data transmission. Reproduced with permission.^[^ ^31^ ^]^ Copyright 2013, American Chemical Society.; H) Scheme of a 3‐input voter and I) a keypad lock.

Compared with above simple logic gates, their combinatorial integration could evolve into advanced logic devices.^[^ ^20^ ^]^ As for the word “computation,” the direct understanding is “arithmetic,” and the most broadly‐used arithmetic logic devices are adders and subtractors, which include half‐adder/subtractor (HA/HS) and full‐adder/subtractor (FA/FS), **Figure** **2**A–D. Take HA for instance, it could achieve the addition of two binary digits. It has two inputs and outputs and relies on the parallel operation of an XOR gate and an AND gate, in which the former executes SUM and the latter plays CARRY functions, respectively. These HA/HS and FA/FS are usually hard to construct at molecular level, because these devices need the parallel operation of logic gates with different functions and the simultaneous production of two distinct signals. Notably, Liu and other scientists designed several HAs with fluorescence outputs based on DNA strand displacement reaction or using DNAzymes as fundamental elements.^[^ ^21^, ^22^ ^]^ Analogously, the HS that composed of XOR and INHIBIT gates was also reported on the basis of Au‐surface immobilized molecular‐beacon system,^[^ ^23^, ^53^ ^]^ in which XOR performed DIFFERENCE and INHIBIT executed BORROW operations, respectively. These works established vivid prototypes for the operation of HA and HS based on facile design and low‐cost DNAs.

Superior to HA/HS that can only calculate 2‐bit operations, FA/FS could perform more complicated 3‐bit calculations, in which FA/FS possess 3 inputs (an additional CARRY‐in for FA and BORROW‐in for FS) and 2 outputs.^[^ ^24^, ^25^ ^]^ And the FA can be obtained via appropriate integration of 2 HAs (2 HSs for FS) and a downstream OR gate, Figure 2C. Molecular FAs/FSs were also achieved by tuning fluorescein's ionization state or by virtue of DNAzyme modules by Willner's group and other scientists.^[^ ^24^, ^25^ ^]^ These adders/subtractors perform indispensable roles in molecular arithmetic functions. In addition to above adders and subtractors, some logic devices (such as arithmetic logic unit (ALU), the most useful devices in digital computer system) integrated the arithmetic/non‐arithmetic logic functions together and worked as arithmetic device overall.

### 2.3 Advanced Logic Devices for Non‐Arithmetic Information‐Processing

Except logic devices for specific arithmetic functions, there are also many ones which are significantly necessary for non‐arithmetic information‐processing, such as encoder/decoder, multiplexer/de‐multiplexer, parity generator/checker, voter, keypad lock, and so on.

Encoders and decoders are widespread non‐arithmetic logic devices in molecular computing as they could achieve the interconversion between binary data and code. Encoders compress binary molecular information for storage or transmission through transforming data signal into code, and decoders just paly the contrary roles to recover the information.^[^ ^26^, ^27^ ^]^ For instance, Gust et al. constructed all‐photonic molecular 4‐to‐2 encoder and 2‐to‐4 decoder based on light‐sensitive organic molecules.^[^ ^27^ ^]^ Although there were no DNAs in this work, this light‐responsive logic device enlightened the design of subsequent DNA‐based encoder/decoder. Xia et al. fabricated series of biomolecular encoders and decoders on a DNA‐based electrochemical biosensor system, Figure 2E, in which the electrochemical signals originated from the interaction between redox‐probe modified DNAs and Au electrodes were taken as outputs.^[^ ^26^ ^]^ Compared with other DNA encoder/decoder systems using fluorescent outputs, this electrochemical approach moved DNA computing a step closer to silicon‐based counterparts.

Unlike encoder/decoders, the multiplexers/de‐multiplexers act as controlled switches and have broad applications in molecular signal processing systems for compressing and decompressing binary information.^[^ ^28^, ^29^ ^]^ Multiplexers are circuits that transform one of multiple input signals into a single output line, and de‐multiplexers perform opposite functions of transforming one input signal into multiple output lines. Notably, Willner's group designed DNA‐based 2:1 and 4:1 multiplexer and a 1:2 de‐multiplexer with DNAzyme‐cleaved fluorescent substrates as output reporters, in which the formation and dissociation of DNAzymes were flexibly controlled by different DNA inputs. One of the potential disadvantages of this work is the frequently‐used dual‐labeled DNA fluorophore‐quencher substrate, which will increase the cost of operation.^[^ ^28^ ^]^ Credi et al. constructed unimolecular 2:1 multiplexer and 1:2 de‐multiplexer using 8‐methoxyquinoline as basic element and corresponding fluorescence signals as different outputs, Figure 2F.^[^ ^29^ ^]^ These works offered typical examples for the design of facile and low‐cost molecular logic systems to construct multiplexers/de‐multiplexers in the future.

Apart from above logic devices for data conversion, the data transmission procedure should also be taken seriously. Through any type of data transmission, the appearance of erroneous bits is an ineluctable phenomenon.^[^ ^30^, ^31^ ^]^ While, these bits can fortunately be detected via putting a parity generator (pG) at sending node and a parity checker (pC) at receiving end, Figure 2G. A 2‐bit even pG (input D1/D2) can yield an additional bit (P) according to an XOR gate's truth table and add P to original bits Dn, varying the number of 1's (*∑*) in the DnP string to even. Then, the generated D1D2P string will be sent to and checked by the 3‐bit even pC. During the normal transmission, the ∑ value of D1D2P string keeps even, accompanying with a “normal” signal (pC produces output “*C* = 0”). While, if errors occur in transmission, the *∑* value of 3‐bit string will be altered to odd and the pC yields output “*C* = 1” as an “alarm.” All the above demonstrates working principle of even pG/pC and the odd one performs similar functions. In 2013, Pischel et al. pioneered the first molecular pG/pC system using photo‐switched organic compound as building block for alerting error in data transmission,^[^ ^31^ ^]^ then molecular pG/pCs were barely reported. Despite this all‐photonic work is pioneering and this versatile photo‐switched organic compound can be used to execute other logic functions, the signal‐to‐noise ratio of the pG/pC device is not sufficiently enough, which should be optimized in future molecular pG/pC systems.

After presenting logic devices for data conversion, compression, and transmission, another functional device we want to introduce herein is molecular voter. The voter is generally derived from Majority logic gate, in which the output will be *TRUE* (1) only in the coexistence of more than half inputs (Proposal “*PASS*”), otherwise it is *FALSE* (0) (“*DENIED*” proposal), Figure 2H. This device could mimic small‐scale voting situations in real life and a typical example of MAJ‐based molecular voter was achieved by Katz's group by exploiting the biocatalytic reactions of different enzymes.^[^ ^32^ ^]^

Protecting molecular information from illegal invasion is the intriguing function that executed by keypad lock. Essentially, the keypad lock is an attractive sequential logic device. Differing from other logic devices, the output of keypad lock depends not only on the presence/absence of inputs, but also their appropriate input sequences (the “password”). Only the correct password could unlock the system, yielding output “1” with “*ON*” state, Figure 2I.

The last interesting logic device for non‐arithmetic information‐processing in this part is the parity‐checker (Pc). Unlike above pG/pC that used for detecting error through data transmission, the Pc could distinguish even/odd natural numbers. During the operation, the decimal numbers are first transformed into 4‐bit binary numbers, by selecting suitable inputs and assigning 4 distinct bits to inputs, the Pc will produce high output “1” in the presence of even (odd) numbers and low output “0” in the presence of odd (even) ones.^[^ ^48^ ^]^ Then the even/odd parity can be easily differentiated.

### 2.4 Other General Cascaded or Concatenated Circuits

**Figure 3:**
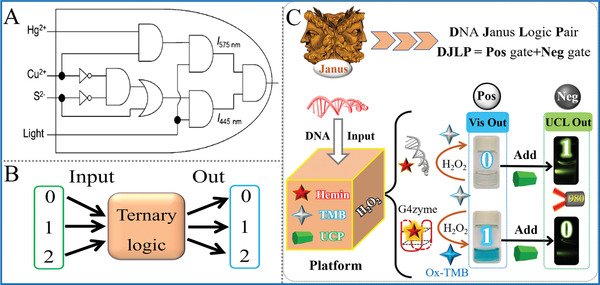
A) A 4‐input concatenated logic circuit, Reproduced with permission‐^[^ ^34^ ^]^ Copyright 2014, Wiley‐VCH; B) Scheme of the ternary logic gate; C) A G‐quadruplex‐based DNA Janus logic pair library, Reproduced with permission.^[^ ^35^ ^]^ Copyright 2019, Royal Society of Chemistry.

As known by molecular engineers, these above logic devices for specific arithmetic logic functions and non‐arithmetic information‐processing are originated from the appropriate cascade or concatenation of common logic gates. However, in some molecular computing systems, the combination of elementary gates will evolve into general cascaded or concatenated circuits without specific functions,^[^ ^33^ ^]^ which possess much more powerful computing ability than any single gate alone. The cascade or concatenation procedure should also comply with the principles of Boolean logic, in which the outputs of upstream logic devices are usually taken as inputs of downstream ones. For example, by exploiting metal‐responsive property of a coumarin–rhodamine conjugate, Jiang et al. operated a 4‐input concatenated logic circuit that triggered by chemical and light inputs, (**Figure** **3**A). This kind of concatenated logic circuit brought largely enhanced computing complexity. More intriguingly, this logic system can also work as a powerful keypad lock.^[^ ^34^ ^]^

Despite the obvious advantages of concatenated circuits over common logic gates, the signal leakage is a non‐negligible problem. Because of the necessary multi‐step operation and the participation of many chemical reactants during cascade or concatenation procedure, the generated circuits usually present general or even low signal‐to‐noise ratio. This phenomenon might bring false‐positive output from upstream logic gate and have severe impacts on downstream ones. To address this issue, exploring novel and optimized system with less (or without) side reaction or byproducts for operating concatenated logic circuits is one of the potential solutions in the future.

### 2.5 Versatile Logic Library

With the rapid advancements of molecular computing, previous logic systems that only execute limited, and specific functions could not meet pluralistic requirements of this area any more. One promising direction is exploring novel and universal system that could operate multifunctional logic devices, termed as “versatile logic library.” One of the famous examples is the Seesaw logic gate system that created by Winfree's group.^[^ ^41^ ^]^ Through delicate design and appropriate TMSD reaction, they achieved scaled‐up digital circuit computing and constructed multilayer logic circuits (including a 4‐bit square‐root calculation), and almost any logic computations can be performed. Interestingly, this concept was also vividly demonstrated by our recent work.^[^ ^35^ ^]^ Based on the luminescence quenching ability of oxidized 3, 3′, 5, 5′‐tetramethylbenzidine (TMB) toward upconversion nanoparticles (UCNPs) and biocatalytic ability of G‐quadruplex DNAzyme, we constructed a universal amphichromatic system that yields double results with half efforts for performing a versatile DNA Janus logic pair library, Figure 3C. In the presence of G4zyme, the generated blue‐colored OxTMB after oxidation could quench the emission of UCNPs via inner‐filter‐effect (IFE), generating the output (1, 0); In the absence of G4zyme, the pale‐blue TMB could not quench the luminescence of UCNPs efficiently, producing output (0, 1). Through reasonable design, a versatile logic library with Janus logic responses was successfully fabricated.

### 2.6 Non‐Boolean Logic (Multivalued Logic, Fuzzy Logic)

All the above logic devices illustrated till now belong to Boolean logic that has 2 input/output states (0/1), whereas binary logic is facing challenges from non‐Boolean logic when processing uncertain or imprecise information. This kind of logic that goes beyond traditional binary operation mainly includes multivalued logic and fuzzy logic.

For the multivalued logic, its each input (output) of possesses more than 2 states (0/1/2…/*n*), bringing obviously increased computing capacities. One kind of simple multivalued logic is ternary logic gate that has 2 inputs and 1 output, in which each input/output exhibits three different states (0/1/2), Figure 3B. Representatively, Qu's group constructed ternary OR and INHIBIT gates based on the assembly of DNA and silicon nanoparticles, demonstrating a typical paradigm for multivalued logic gates.^[^ ^36^ ^]^

For fuzzy logic, it uses analogue region to implement “soft” computation for dealing with uncertain information. The fuzzy variable that undergoes fuzzy sets is taken as values, and the linguistic variables (such as low, medium, high) are applied as corresponding outputs. The result of fuzzy logic usually can be concluded via *IF‐THEN* procedure. For instance, Huang et al. built a fuzzy logic tree through a graphene‐based chemical system and took it as an intelligent molecular searcher.^[^ ^37^ ^]^

To give a brief summary, we discussed the typical categories, detailed functions and operating principles of various logic devices in this section. It should be noted that these described logic devices are just the tip of an iceberg among the abundant “logic‐toolbox.” Under scientists’ continuous efforts, it is anticipated that more and more logic devices with innovative functions can be designed.

## 3 DNA Logic Systems Based on Multifarious “Building‐Block” Materials

**Figure 4:**
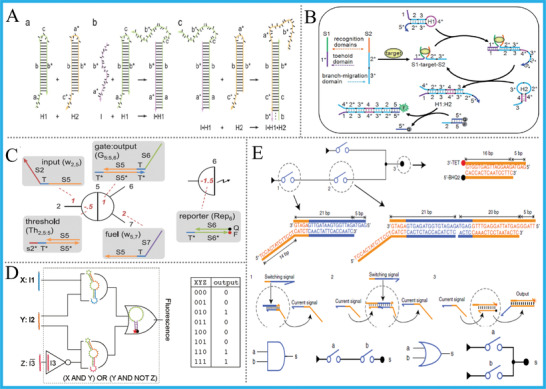
A) Mechanism of the HCR, Reprinted with permission.^[^ ^38^ ^]^ Copyright 2004, National Academy of Sciences; B) CHA reaction‐based DNA logic system, Reproduced with permission.^[^ ^40^ ^]^ Copyright 2015, Royal Society of Chemistry; C) TMSD‐based “seesaw” gate, Reproduced with permission.^[^ ^41^ ^]^ Copyright 2011, American Association for the Advancement of Science; D) DNA‐based reversible logic gate, Reproduced with permission.^[^ ^42^ ^]^ Copyright 2011, American Chemical Society; E) Switching circuit‐based DNA logic system, Reproduced with permission.^[^ ^43^ ^]^ Copyright 2020, Springer Nature.

With the surprising evolution of DNA technology that inaugurated by Ned Seeman, varieties of DNA‐based computing platforms have been delicately designed, in which DNA hybridization is the central issue. Apart from the simple hybridization, the classical and well‐established toehold‐mediated strand displacement (TMSD), derived hybridization chain reaction (HCR)^[^ ^38^, ^39^ ^]^ and catalytic hairpin assembly (CHA)^[^ ^40^ ^]^ are widely employed to DNA logic computing and amplified biosensing, **Figure** **4**A,B. During the TMSD reaction, two DNA strands hybridize with each other to form the duplex, in which the longer one (strand L) has the exposed toehold. In the presence of another strand (strand I) that is almost fully complementary to L, I will react with the toehold first and initiate the branch immigration reaction and displace the short strand, finishing the TMSD reaction with a duplex L/I.

Representatively, Winfree et al. created a TMSD‐based “seesaw” gate motif and used it to construct enzyme‐free large‐scale digital logic circuitry in the pioneering work, thereinto a sophisticated 4‐bit square‐root calculation was appropriately performed, Figure 4C.^[^ ^41^ ^]^ And it should be noted that this “seesaw” gate system can theoretically perform any logic computations. Turberfield et al. achieved DNA reversible logic circuits based on TMSD and a unique DNA hairpin structure, and the circuits show kinetic and thermodynamic reversibility that respond nonlinearly to inputs’ contents, Figure 4D.^[^ ^42^ ^]^ This work inspired the operation of DNA reversible logic gates and DNA nanomachine with reconfigurable property. Recently, Fan's group combined TMSD with DNA switching circuits (DSCs) together and realized a set of DSCs‐based digital computing functions (such as full‐adding), Figure 4E.^[^ ^43^ ^]^ They demonstrated that arbitrary Boolean functions can be implemented by DSCs with high computing speed, thus providing an inspiring paradigm for biomolecular computing. All above works depend only on the innovation of DNA hybridization technology, the TMSD reaction and fluorophore‐labeled DNAs, not on functional materials, which act the leading and enlightening role in DNA computing.

In addition to above TMSD‐related digital computing works, many DNA logic systems using different “toolbox” materials as substrates are also reported, which we will survey in this section and is also theme of this review. Notably, some logic systems integrate the advantages of more than one kind of material, and when we conduct this review, the characteristics of selected representative examples in each type of “building‐block” material are alternatively described. We mainly summarize related state‐of‐the‐art works of each category in recent 5 years, whereas some classical/famous ones reported earlier are also included.

### 3.1 Functional DNA Motifs

#### 3.1.1 Aptamers

Aptamers are short DNA/RNA strands that selected via SELEX procedures.^[^ ^44^ ^]^ The charming property is that they could bind target ligands with high‐affinity, and have been extensively applied to bioanalysis, target capture/isolation, DNA computing, and other intriguing areas. The combination of aptamer and logic could not only enrich DNA computing systems, but also enable the logic‐guided smart analysis of targets or even monitoring the cellular functions specifically. And the widely‐used aptamers in DNA computing are anti‐ATP and anti‐thrombin aptamers.

**Figure 5:**
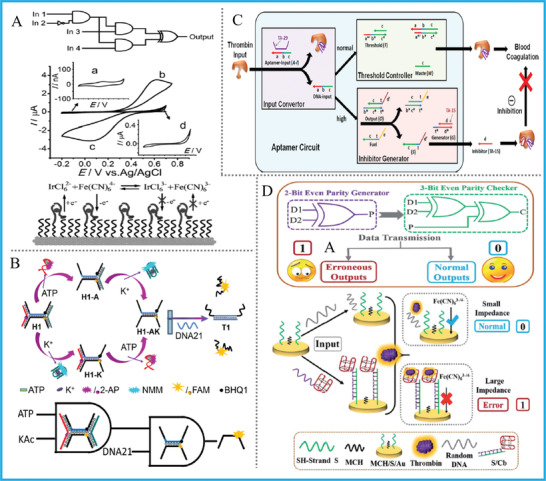
A) ATP/ABA‐based multi‐level logic circuit, Reproduced with permission.^[^ ^45^ ^]^ Copyright 2015, Wiley‐VCH; B) ATP/ABA‐based cascaded logic circuit, Adapted with permission.^[^ ^46^ ^]^ Copyright 2018, Elsevier; C) Thrombin/TBA‐based logic system for modulating protein activity, Reproduced with permission.^[^ ^47^ ^]^ Copyright 2012, American Chemical Society; D) Thrombin/TBA‐based DNA pG/pC system, Reproduced with permission.^[^ ^48^ ^]^ Copyright, 2018, Royal Society of Chemistry.

For ATP and its aptamer ABA, by taking advantage of split‐ABA, ATP, adenosine deaminase (ADA that catalyzes ATP's deamination), and electrochemical rectification, Feng et al. fabricated multi‐level logic gates (INH, INH‐AND, and INH‐AND‐XOR) which realized total XOR‐guided bioanalysis of physiological level of ATP/ADA, and showed the potential of distinguishing health statuses or accurate diagnosis, **Figure** **5**A.^[^ ^45^ ^]^ Despite the largely improved diagnostic significance of above multi‐level circuits, the complicated preparation of the electrode and fabrication of rectification electrochemical system is a non‐negligible problem. Besides, Li's group constructed two cell‐environment‐responsive cascaded “AND‐AND” and “OR‐AND” logic circuits with fluorescent responses, in which ATP, ABA, K^+^, and G‐quadruplex were reasonably integrated into one H‐shaped DNA nanostructure for controllable molecular sensing, Figure 5B.^[^ ^46^ ^]^ Compared with above electrochemical system, this optical one can be facilely designed and realize fast logical analysis of different targets.

For DNA logic systems based on thrombin and its aptamer TBA, a famous work was presented by Tan's group previously. They built the first logic circuit that possesses accurate threshold control on the basis of DNA/protein interactions, which realized automatic, self‐sustained, and logic‐guided modulation of thrombin activity in vitro, Figure 5C.^[^ ^47^ ^]^ The circuit could sense the local content of thrombin, and autonomously release a coagulation inhibitor at extraordinary high concentration of target. Remarkably, our group designed the first electrochemical DNA parity generator/checker for error detection in data transmission, Figure 5D.^[^ ^48^ ^]^ TBA was integrated into the ends of input‐strands and acted as “nanoclaw” to capture thrombin, and electrochemical impedance signal changes modulated by “aptamer‐nanoclaw” were used as corresponding outputs. This work inspired the operation of novel electrochemical logic devices and logic‐directed target detection based on the interaction between aptamer and targets. Besides, DNA computing platforms fabricated upon other DNA aptamers that targeted for PDGF‐BB protein, antibiotics, and even RNA aptamers were also reported.^[^ ^49^, ^50^, ^51^ ^]^

#### 3.1.2 Metal‐Ion Dependent DNAzyme or DNA Nanostructure

Metal‐ion dependent DNAzymes (or deoxyribozymes) are functional nucleic acids that could catalyze the cleavage of ribonucleobase‐modified substrate DNA strand in the presence of specific metal ions. This versatile “catalytic enzyme” was created by Breaker in 1994,^[^ ^52^ ^]^ since when plenty of optical and electrochemical biosensors and biocomputing systems relying on different metal‐ion DNAzymes were successively reported.

**Figure 6:**
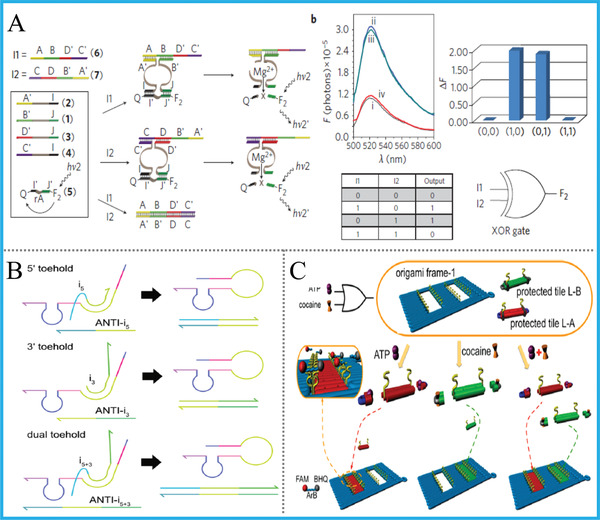
A) Scheme of XOR gate based on the Mg^2+^‐dependent DNAzyme. Reproduced with permission.^[^ ^53^ ^]^ Copyright 2010, Springer Nature; B) Operation of the toehold‐integrated Mg^2+^‐DNAzyme for repeated DNA logic gate. Reproduced with permission.^[^ ^55^ ^]^ Copyright 2019, American Chemical Society; C) ATP/cocaine triggered Mg^2+^‐DNAzyme for logical pattern of DNA origami, Reproduced with permission.^[^ ^56^ ^]^ Copyright 2016, American Chemical Society.

Notably and impressively, Willner's group constructed a universal DNA computing platform by utilizing a set of Mg^2+^‐dependent DNAzymes.^[^ ^53^ ^]^ Under the input‐directed assembly, the formation and dissociation of DNAzyme can be selectively controlled, and a library of versatile logic devices, including common logic gates, half‐adder/‐subtractor, cascaded multilayer circuits, and simultaneous gates’ operation were all successfully accomplished, **Figure** **6**A. On the basis of the similar system, pH‐responsive DNA logic arrays propelled by Mg^2+^/UO_2_^2+^ DNAzyme units were further realized.^[^ ^54^ ^]^ Recently, Macdonald's group smartly integrated TMSD reaction and the catalytic property of DNAzymes together, and achieved the repeated use of DNA logic gates.^[^ ^55^ ^]^ The DNAzyme‐based logic units can be controllably and cyclically activated while maintaining 90−125% of original performance, and the amount of redundant DNA strands can be reduced, obviously increasing the computing efficiency, Figure 6B. Besides, to achieve more advanced logic functions, Milan N Stojanovic & Darko Stefanovic also did an impressive work on DNA logic computation with DNAzymes in 2003.^[^ ^14^ ^]^ In their system, a molecular automaton called MAYA was constructed, which could encode a version of the game of tic‐tac‐toe and interactively competes against a human opponent. Except the simple DNA nanostructures, DNAzymes were also elegantly introduced into DNA origami or other functional material‐based systems to perform logical operations, Figure 6C.^[^ ^56^ ^]^

In addition to DNAzymes, some metal ions were found to have unique interactions with DNA bases, such as the well‐known T‐Hg^2+^‐T, C‐Ag^+^‐C base pairing.^[^ ^57^ ^]^ For instance, by taking advantage of Ag^+^, Hg^2+^, their reaction with Cysteine/GSH and target‐responsive mesoporous silica nanocontainers, Qu's group designed a versatile DNA logic system with peak current intensity as output signals.^[^ ^57^ ^]^ Additionally, there are also DNA computing systems performed upon other metal‐ion‐triggered DNA nanostructures, such as K^+^/CE (crown ether)‐modulated formation of G‐quadruplex, Ag^+^ triggered i‐motif, and so on, which will be alternatively discussed in next sections.

#### 3.1.3 G‐Quadruplex

One kind of famous non‐canonical DNA secondary structures is G‐quadruplex. Generally, four guanine bases can form a G‐quartet through Hoogsteen‐base paring and the stacking of multiple G‐quartets generates complete G‐quadruplex (G4) in the presence of metal cations (K^+^, Na^+^, or Sr^2+^).^[^ ^58^, ^59^ ^]^ And its formation/dissociation can be reversibly switched by corresponding chelating agent, such as K^+^/CE or Sr^2+^/KP (kryptofix [2.2.2]) pairs, and this phenomenon exhibits promising potential for flexible DNA computing. Owing to multifarious fascinating merits, G4 is considered to be one of the most popular functional DNA motifs, and two kinds of well‐known properties of G4 have been extensively used to operate versatile DNA logic devices. On the one hand, G4 could bind with porphyrin dye via end‐stacking effect and dramatically enhance its fluorescence. On the other hand, it has been confirmed that the stacking mixture of G4/hemin (G4zyme) possess efficient peroxidase‐mimicking property, which catalyzes the oxidation of various colorimetric or fluorescent substrates by H_2_O_2_.^[^ ^60^ ^]^

**Figure 7:**
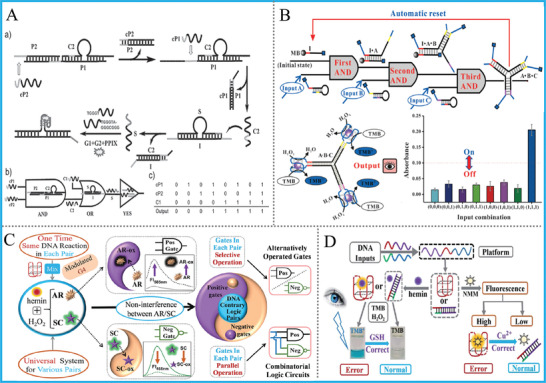
A) AND‐OR‐YES cascaded circuit based on TMSD‐modulated G4/PPIX. Reproduced with permission.^[^ ^59^ ^]^ Copyright 2013, Wiley‐VCH; B) Concatenated logic circuit based on CHA‐triggered formation of G4zyme. Reproduced with permission.^[^ ^60^ ^]^ Copyright 2015, Wiley‐VCH; C) Schematic operation of the G4zyme‐based “contrary logic pairs” (CLPs) library. Reproduced with permission.^[^ ^62^ ^]^ Copyright 2017, Royal Society of Chemistry; D) Operation of the DNA‐based pG/pC system. Reproduced with permission.^[^ ^63^ ^]^ Copyright 2017, Royal Society of Chemistry.

Notably, in our early work, we combined DNA TMSD reaction with split‐G4 modulated fluorescence together, and realized a series of label‐free and non‐enzymatic DNA logic circuits, and logic‐controlled load/release of the drug model PPIX (protoporphyrin IX) was also proposed, **Figure** **7**A.^[^ ^59^ ^]^ For the G4zyme‐based computing system, Chen et al. constructed a keypad lock system with visual readout and automatic‐reset function by harnessing three‐way‐junction‐based hairpin assembly, magnetic separation, and the catalytic oxidation of TMB by H_2_O_2_. Figure 7B.^[^ ^60^ ^]^ Analogously, our group took advantage of split‐G4zyme, elegant sequence design and the dissociation ability of Cu^2+^ toward the structure of 24GT, realized the first DNA‐based voter with visual out and “one‐vote deny” function. Besides, the OR‐INH cascaded logic circuit and IMP gate were further achieved.^[^ ^61^ ^]^ Despite the smart design, the high background signal originated from the formation of different parts of half G4 was a potential problem, which was generally solved via accurate optimization of the inputs’ concentration.

In addition to colorimetric substrates, fluorescent ones of G4zyme were also used to DNA computing. Innovatively and interestingly, our group reported an intelligent universal system that “kills two birds with one stone” for engineering a DNA “Contrary Logic Pairs” (CLPs) library and various DNA combinatorial logic circuits by exploiting two fluorescent substrates (Scopoletin, Amplex Red) of G4zyme with inverse responses as label‐free output‐reporters. Figure 7C.^[^ ^62^ ^]^ The “Pos^Neg” gates of each DNA CLP in this system were constructed via the same once DNA reaction, avoiding gates’ redesign/reoperation in previous works. This work evidently simplified the operation and reduced the costs/time of DNA gates by at least half, accompanied by prominently elevated computing efficiency. Apart from abovementioned works, the simultaneous use of G4's two kinds of properties for DNA computing was also proposed. Our group established a DNA‐based parity generator/checker (pG/pC) for error detection in data transmission with input‐regulated split‐G4/‐G4zyme as signal reporters. Figure 7D.^[^ ^63^ ^]^ Superior to previous pG/pC systems, this one can exhibit not only fluorescence outputs but also facile visual ones that distinguished by naked eye, and multi‐level concatenated logic circuits with dual output‐mode were further achieved via appropriate design. To the best of our knowledge, this was the first versatile DNA pG/pC system, which can be used for error detection through DNA‐based data transmission.

Of note, there are also some DNA logic platforms that relying on the combination of G4 with labeled‐probes or other organic dyes, but are not fully included herein. These works further boosted the developments of innovative DNA computing paradigms.

#### 3.1.4 I‐Motif

After demonstrating the DNA logic platforms with G4 as building elements, one may realize that there is more than one type of tetraplex DNA structures. Similar to G4, i‐motif is composed of poly‐cytosine sequences and can be stabilized by proton (H^+^) via forming hemi‐protonated C‐C^+^ base pair, and it plays significant roles in many biological functions. For i‐motif‐based DNA computing, some works reported the operation of basic DNA logic gates by selectively illuminating specific organic dyes (such as crystal violet) under diverse input states.^[^ ^64^ ^]^ Notably, Yang's group took advantage of tetraplex structures and two unique dyes (*Nap‐s* and *Ben‐eth*) as signal‐reporters and established a completely label‐free logic system, in which adders, subtractors, majority and dual‐transfer gates were exquisitely realized,^[^ ^65^ ^]^ They further used this system as smart and selective biosensor to analyze the disease‐related miRNAs. Although these dyes presented satisfied signal‐to‐noise ratio, they usually need long‐time synthesis and sophisticated purification/separation steps. Exploring low‐cost, commercially‐available dyes that could specifically bind i‐motif is one of the potential solutions.

**Figure 8:**
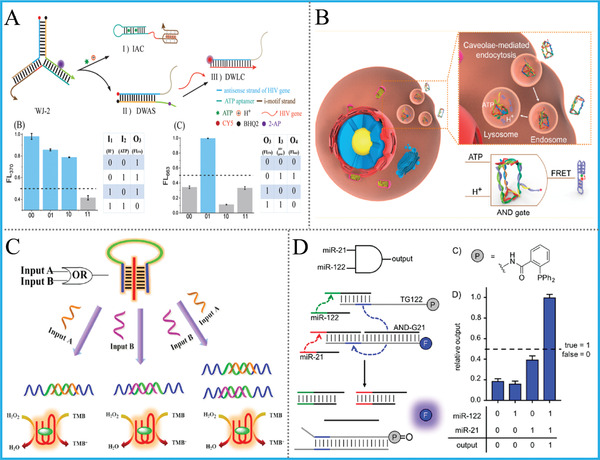
A) Tandem logic circuits based on i‐motif, ATP, pH and DNA 3‐WJ. Reproduced with permission.^[^ ^66^ ^]^ Copyright 2018, Royal Society of Chemistry; B) Introduction of i‐motif and ATP aptamer into framework DNA for subcellular imaging. Reproduced with permission.^[^ ^67^ ^]^ Copyright 2019, American Chemical Society; C) MB‐caged G‐quadruplex for operating colorimetric DNA logic gates. Reproduced with permission.^[^ ^70^ ^]^ Copyright 2014, American Chemical Society. D) DNA logic‐controlled release of small molecules. Reproduced with permission.^[^ ^71^ ^]^ Copyright 2017, American Chemical Society.

Additionally, Li's group took i‐motif as structure‐controlling elements and incorporated HIV gene, ATP and pH value with a universal three‐way junction system, and the tandem AND‐AND, NAND‐INH logic circuits were alternatively realized, **Figure** **8**A.^[^ ^66^ ^]^ Analogously, they introduced a lysosome‐recognizing DNA framework device into cancer cells for logic‐controlled subcellular imaging by utilizing i‐motif and ATP aptamer as intracellular target‐responsive bridging units, Figure 8B.^[^ ^67^ ^]^ This solid work opened up inspiring avenues for logic‐guided intracellular imaging and drug‐release based on functional DNA motifs.

#### 3.1.5 Triplex, Molecular Beacon and Artificially‐Modified DNA

Except the frequently‐used duplex and tetraplex, other functional DNA motifs, such as triplex, hairpin‐based MB and DNA strands with artificially‐modified bases were further exploited to DNA computing. The triplex structure that depends on the synergistic effect of Watson‐Crick and Hoogsteen interactions, includes classical T·A‐T and C·G‐C^+^ pairs, wherein the former is stable at neutral pH but will dissociate into duplex and single‐strand under basic conditions, and the latter one keeps stable at acid pH but undergoes disassembly when the environment is neutral. To give an example, Pei's group used metal‐ion triggered assembly of C·G‐C^+^ triplex to flexibly control the formation of G4 and constructed AND, OR, INH gates with the fluorescence of N‐methyl mesoporphyrin IX (NMM) as outputs.^[^ ^68^ ^]^

For the hairpin‐derived MB, the fascinating merits of this structure are its high flexibility, low background, and elimination of tedious nano‐quenchers. A famous example of MB‐based logic computing was demonstrated by our group in 2015.^[^ ^69^ ^]^ By integrating a universal fluorophore/quencher‐labeled MB with G4/NMM system, Li et al. constructed a library of advanced arithmetic logic devices, including HA/HS, FA/FS, and a digital comparator. The obvious advantage of this work over previous ones is multiple output signals can be concomitantly generated while sharing the same operating platform, but it will be a better system if the labeled fluorophores can be replaced by other non‐covalent probes. Notably, the simultaneous use of triplex and MB for colorimetric DNA logic gates was also achieved with triplex‐caged G4zyme as output‐generator, Figure 8C.^[^ ^70^ ^]^

In addition to above stimuli‐responsive DNA nanostructures, some researchers pay attention to modifying DNA bases with functional organic molecules (not only organic fluorophores) to obtain artificial DNA strands, and further introduce these strands to DNA computing. Especially, Deiters’ group synthesized several fluorophore‐azido‐, phosphine‐, and other organic molecules modified oligonucleotides, then they designed miRNA‐triggered AND/OR DNA logic gates by exploiting Staudinger reduction as basic principle for the controlled release and activation of small molecule fluorophores, Figure 8D.^[^ ^71^ ^]^ Furthermore, they “wired” these gates in cascade to generate more sophisticated logic circuits that stimulated by three distinct miRNAs. The versatile oligonucleotides‐modification techniques used in this work might boost the advancements of DNA technology and find applications in DNA‐based catalysis/synthesis and so on. Besides, this work enlightened the potential of DNA computing to interact with biomarkers under chemical or cellular‐mimicking environments for potential promising bio‐applications.

### 3.2 DNA Tool‐Enzymes

**Figure 9:**
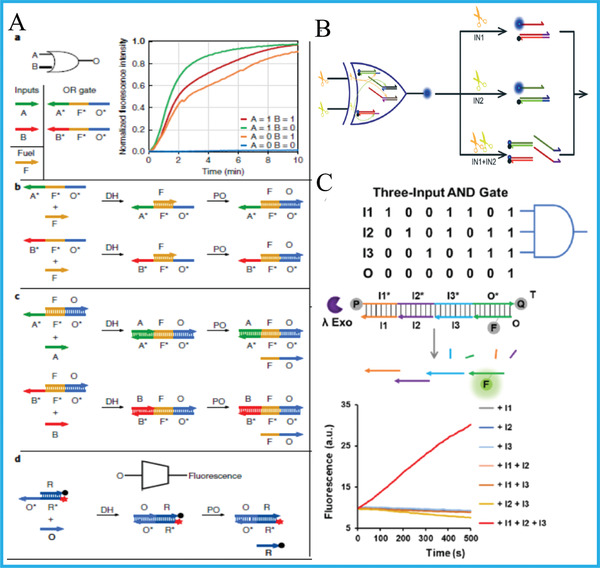
A) OR gate based on polymerase‐induced strand displacement. Reproduced with permission.^[^ ^72^ ^]^ Copyright 2019, Springer Nature; B) XOR gate based on two kinds of nicking enzymes. Reproduced with permission.^[^ ^74^ ^]^ Copyright 2019, Royal Society of Chemistry.; C) 3‐input AND gate based on *λ*‐exonuclease. Reproduced with permission.^[^ ^75^ ^]^ Copyright 2020, Royal Society of Chemistry.

Although enzyme‐free DNA logic devices dramatically reduced the operating cost and time, the participation of miscellaneous DNA tool‐enzymes (such as polymerase, nicking enzyme, exonuclease, etc.) in DNA computing can not only lower the leaking ratio of output signals, but also evidently improve the computing flexibility and efficiency. Recently, by utilizing strand‐displacing DNA polymerase and single‐stranded gates, Song et al. innovatively reported the operation of a set of DNA logic circuits, including elementary gates, multilayer cascaded circuits, moderate, and large scale circuits with unique functions, **Figure** **9**A.^[^ ^72^ ^]^ There were only single DNA strands in this system, which obviously reduced the signal leakage/restoration steps and the number of used DNA strands, such that the computing speed and efficiency was vividly improved. Similarly, to overcome the restrictions of poor integration efficiency, limited computing functions of DNA computing, Zhou's group constructed a DNA arithmetic logic unit (ALU) consisting of common DNA logic gates using the similar polymerase‐induced strand displacement.^[^ ^73^ ^]^ The use of enzymes brought highly efficient logic gates which are suitable for multiple cascades. Their work offers a facile method for assembling large‐scale DNA computing system, enlightening the great potential for controlling the molecular behaviors of complicated biosystems.

Different from polymerase, nicking enzymes could cleave specific sequences of DNA strands with satisfied accuracy and also have been applied to DNA computing. For instance, Zhao et al. exploited two carefully selected nicking enzymes (Nb.BtsI and Nt.BbvCI) to build a platform for executing HA and HS with acceptable signal‐to‐noise ratio, Figure 9B.^[^ ^74^ ^]^ Similar to nicking enzymes, exonuclease could also digest nucleotides with high‐efficiency. Su's group incorporated TMSD reaction, dual‐site binding approach and *λ*‐exonuclease into one system and operated multi‐layer cascaded circuit (AND‐OR‐AND), Figure 9C.^[^ ^75^ ^]^ In addition, Shapiro's group constructed an autonomous molecular computer which could achieve logical control of gene expression, in which the restriction nuclease was used as hardware molecules.^[^ ^76^ ^]^ This molecular computer could logically analyze the levels of messenger RNA species, and generate a molecule capable of changing gene expression. This work has the potential of being applied to bioanalysis, genetic engineering, and diseases diagnosis.

### 3.3 Non‐DNA Biomaterials

**Figure 10:**
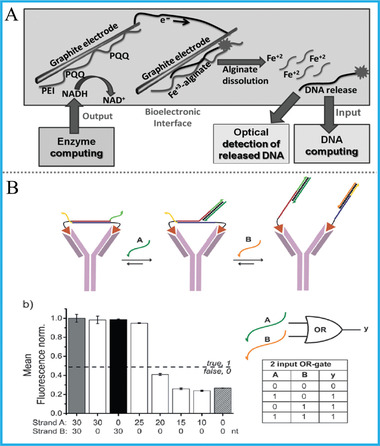
A) A universal interface that bridges enzymatic computing systems and DNA ones. Reproduced with permission.^[^ ^77^ ^]^ Copyright 2015, Wiley‐VCH; B) OR gate based on antibody/DNA interactions. Reproduced with permission.^[^ ^79^ ^]^ Copyright 2014, Wiley‐VCH.

Non‐DNA biomaterials, include natural enzymes, proteins, antibodies, and so on, are broadly used in bio‐catalysis, bio‐analysis, controlled gene expression, and other valuable areas. Their involvements in molecular/DNA computing will not only enrich the logic systems, but also bring excellent biocompatibility and targeting ability. Typically, Katz's group developed a universal interface that bridges the natural enzyme‐based computing systems and DNA ones to achieve their connection, **Figure** **10**A.^[^ ^77^ ^]^ The upstream enzyme system used small molecules as inputs and generated NADH as output after various enzyme‐based catalytic reactions, which further stimulated electrochemical release of a DNA strand. Then this strand will be received by downstream DNA system and execute DNA computing tasks.

For the combination of protein with DNA computing, Deiters's group designed AND, OR, and NOR logic gates by utilizing zinc‐finger proteins, in which these DNA‐triggered gates could control the activation/deactivation of split‐luciferases and generate a protein output.^[^ ^78^ ^]^ Furthermore, microRNAs were applied to execute logic‐guided protein activation. In addition, antibody/antigen pairs play a pivotal role in enzyme‐linked immunosorbent assay (ELISA) and point‐of‐care (POC) analysis. For their application in DNA computing, Merkx's group did many novel and interesting works based on antibody‐templated strand displacement (ATSD). They used bivalent peptide–DNA conjugates as non‐covalent molecular locks to execute OR or AND logic‐programmed antibody activity, Figure 10B.^[^ ^79^, ^80^ ^]^ And the reversible regulation of antibody targeting was realized with only nm DNA‐peptide locks. Although this work provides an efficient technique for modifying peptide on DNA strands, one of its non‐negligible problems is that the modification of DNA on peptide needs multi‐step separation and purification, which might result in the time wastage and increase of cost.

To give a brief summary, all of above elegant works that integrated both merits of non‐DNA biomaterials and DNA logic, which paved unique ways and laid solid foundations for the application of DNA computing to intelligent bioanalysis, diagnosis, and therapy.

### 3.4 Multifarious Nanomaterials or Polymers

Apart from biomaterials‐based DNA computing systems, the striking developments of colorful and multifarious nanomaterials or polymers will undoubtedly introduce strengthened flexibility, diversity, and versatility to this field. In this section, we discuss DNA computing systems built upon varieties of nanomaterials, including gold nanoparticles (AuNPs), magnetic beads, carbon nanomaterials (graphene oxide, polydopamine nanosphere, and carbon nanotube), DNA‐templated metal nanoclusters, other luminescent nanomaterials, and even polymers, in which some representative works are selectively illustrated.

#### 3.4.1 AuNPs

AuNPs are widely applied to many different research areas, this is the reason why we would like to introduce it first. The combination of AuNPs with DNA has drawn considerable and widespread interests and exhibited crucial power in various applications, such as DNA‐based visual detection of targets,^[^ ^81^ ^]^ binding‐induced DNA nanomachine for amplified biosensing^[^ ^82^ ^]^ and versatile DNA computing. Considering that there are many AuNPs‐based DNA computing systems, we herein just demonstrate representative examples.

**Figure 11:**
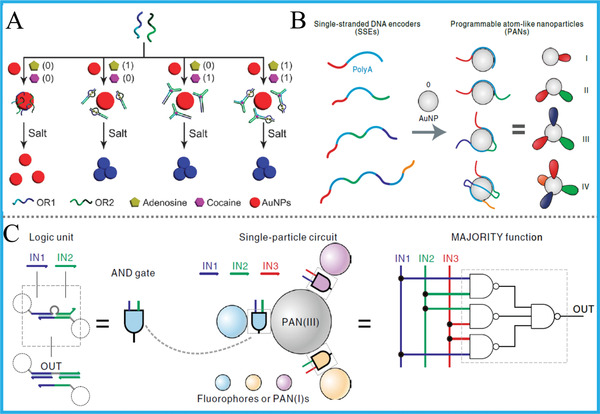
A) DNA OR gate based on target/aptamer induced AuNPs aggregation. Reproduced with permission from.^[^ ^81^ ^]^ Copyright 2011, Royal Society of Chemistry; B) Illustration of the programmable atom‐like nanoparticles PANs and C) corresponding operation of DNA logic gates. Reproduced with permission.^[^ ^85^Copyright 2019, Springer Nature.

Most of reported works mainly rely on the distinctive adsorption ability of naked AuNPs toward duplex and single‐strand DNA (ssDNA). For the interacting mechanism, the ssDNA can be facilely adsorbed on the surface of AuNPs after uncoiling its partial bases and prevent salt‐induced aggregation. On the contrary, dsDNA has more rigid structure and exposes phosphate backbone with negative charge, resulting in weak binding and subsequent salt‐induced aggregation of AuNPs. For instance, Yang’ group constructed colorimetric AND and OR gates with direct visual outputs by making use of split/complete aptamer of ATP/cocaine and pure AuNPs, **Figure** **11**A.^[^ ^81^ ^]^ Similarly, Wang's group took advantage of AuNPs, bisphenol A/S and their aptamer, realized cascaded DNA logic circuits (IMP/IMP‐OR) for concomitant target detection.^[^ ^83^ ^]^ Compared with the non‐covalent interaction, the covalent modification of DNA on AuNPs could achieve more reliable and accurate control of DNA hybridization, bringing satisfied signal‐to‐noise ratio to logic computing systems. For the covalent modification method, some works also used SH‐DNA modified AuNPs for DNA computing. Wang's group fabricated a library of strip biosensor logic operations by combining streptavidin‐biotin interaction, protein‐aptamer binding and aptamer‐attached AuNPs, and three different proteins can be distinguished by the naked eye.^[^ ^84^ ^]^

Additionally, another work we want to emphasize is the programmed multi‐valence polyadenine (poly‐A)/AuNPs DNA encoder that reported by Fan's group not long ago.^[^ ^85^ ^]^ Different from abovementioned ones, poly‐A region was taken as the non‐covalent linker. They explored a method for patterning colloidal gold nanoparticles with valence variety using ssDNA encoders containing poly‐A. By controlling the property of each encoder with regulated poly‐A/non‐poly‐A regions, Figure 11B,C, they synthesized programmable atom‐like nanoparticles (PANs) and established a set of Boolean logic devices, including AND, XOR, OR, NAND, XNOR, NOR, and MAJORITY gates. This solid work inspired the design of molecular logic devices and accurate control of biological functions based on single‐particle nanotechnology.

#### 3.4.2 Magnetic Beads

**Figure 12:**
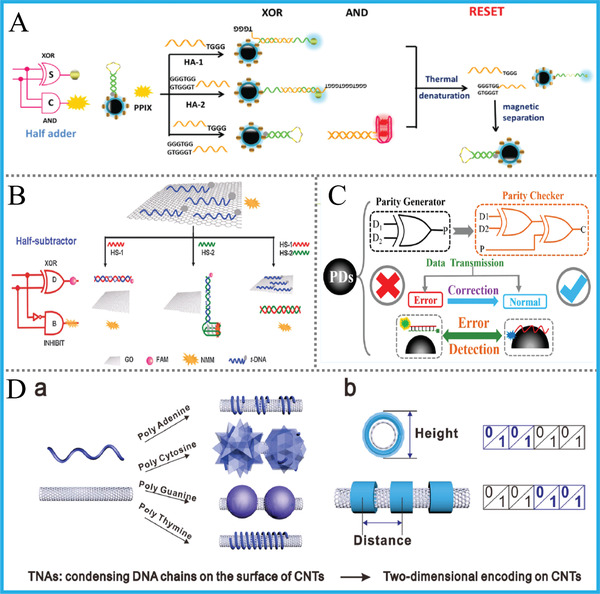
A) The operation of resettable HA based on MB. Reproduced with permission.^[^ ^87^ ^]^ Copyright 2015, Royal Society of Chemistry; B) GO‐based HS by combining G4 with FAM‐DNA. Reproduced with permission.^[^ ^88^ ^]^ Copyright 2014, Royal Society of Chemistry; C) Operation of the PDs‐based pG/pC system. Reproduced with permission.^[^ ^90^ ^]^ Copyright 2017, American Chemical Society; D) CNTs‐based DNA data storage platform. Reproduced with permission.^[^ ^91^ ^]^ Copyright 2019, American Chemical Society.

Owing to the inherent unique property of magnetic separation, magnetic beads (Fe_3_O_4_ nanoparticles, Magnetic beads [Mbs]) have been integrated with various biomolecules to achieve target enrichment/separation, ELISA, POCT, and so on. The first application of Mbs to DNA computing was demonstrated by Adleman in 1994, which was also the pioneering work of this area.^[^ ^19^ ^]^ In DNA computing, the use of Mbs could achieve the efficient separation of product and byproducts, bringing obviously improved signal‐to‐noise ratio and also novel logic functions. Similarly, by making use of this property and catalytic hairpin assembly, Chen's group implemented a DNA keypad lock system with auto‐reset function and multi‐input concatenated circuit was further implemented.^[^ ^60^ ^]^ Inspired by above works, Zhang et al. constructed series of advanced arithmetic and non‐arithmetic logic devices (such as HA/HS, encoder/decoder) with reconfigurable and resettable features via magnetic separating and heating the computing modules, **Figure** **12**A.^[^ ^86^, ^87^ ^]^ These works endowed DNA computing innovative functions and also improved the utilization efficiency.

#### 3.4.3 Carbon Nanomaterials: Graphene Oxide, Polydopamine Nanospheres, and Carbon Nanotubes

Carbon based or related nanomaterials were extensively used in fluorescent biosensing, electrochemical analysis and electrocatalysis. Their application in DNA computing mainly depends on the fluorescent quenching ability toward fluorophore‐attached DNA after trapping it on the surface. Owing to the excellent absorbing ability of carbon nanomaterials toward ssDNA and sufficient fluorescence quenching ability toward fluorophores or dyes, their participation in DNA computing usually could generate satisfied output signals. Herein, we will introduce DNA computing systems based on graphene oxide (GO), polydopamine nanospheres (PDs), and carbon nanotubes (CNTs) as representatives.

GO, a kind of 2D nanomaterial with unique optical, electronic, and catalytic merits, has been confirmed to be superior building blocks for bioelectronic devices. According to previous reports,^[^ ^88^ ^]^ GO could quench the fluorescence of different dyes via long‐range resonance energy transfer and distinguish varying DNA structures. Through *π*—*π* stacking interaction, ssDNA can be adsorbed on GO and set into freedom upon forming duplex or secondary structures. By exploiting this property and elegantly incorporating it with G4/porphyrin dye or fluorophore‐labeled DNAs, our group constructed lots of enzyme‐free DNA logic devices, including basic gates, HA/HS (Figure 12B), multiplexer/demultiplexer, and so on.^[^ ^89^ ^]^

PDs and its derivatives are the polymerization product of monomer dopamine with excellent biocompatibility. They were widely applied to diverse biological areas or as coating material/precursor of other nanomaterials. Taking advantage of the similar luminescent quenching ability of PDs to GO, we for the first time applied this material to DNA computing and designed a label‐free and G4‐free DNA pG/pC system for error detection in data transmission, Figure 12C.^[^ ^90^ ^]^ The implementation of this system can be finished within 1 h in a simple and cost‐effective way, and 3‐input concatenated XOR‐INH circuit was also achieved.

In most cases, DNA computing systems based on nano‐quenchers were operated upon their luminescent quenching ability, whereas the adsorption property toward DNA was not taken seriously. Coincidently, Zuo's group developed a novel kind of tubular nucleic acid (TNA) via condensing DNA strands on the surface of 1D CNTs, Figure 12D.^[^ ^91^ ^]^ The TNAs presented distinct patterns with unique height/distance that can be applied to 2D encoding on CNTs and also to information storage with direct visual output. Although this innovative work that implemented without DNA hybridization offered a cost‐effective strategy to DNA‐based molecular data storage, the specific synthesis conditions and long reaction time are the limitations. Exploring fast and facile DNA‐based data storage system remains further endeavors in the future.

#### 3.4.4 DNA‐Templated Metal Nanoclusters

DNA‐templated metal nanoclusters are composed of a few to a hundred of metal atoms that obtained via in situ reduction of metal ions with specific DNA strand as template (such as poly‐C based silver nanocluster). Benefiting from the facile synthesis and adjustable fluorescent emission, DNA‐templated nanoclusters have been efficient and intriguing luminophores and attracted considerable attentions from various areas. The introduction of them to DNA computing will not only avoid expensive and tedious modification steps, but also greatly reduce the cost and complexity of labeled‐probes.

**Figure 13:**
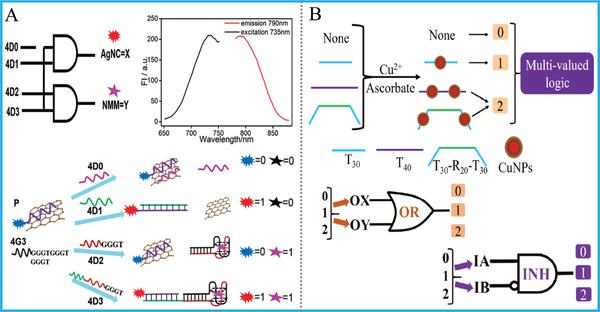
A) The 4‐to‐2 encoder based on the assembly of GO and DNA‐AgNCs. Reproduced with permission.^[^ ^92^ ^]^ Copyright 2016, Royal Society of Chemistry; B) Operation of the ternary logic gate based on DNA‐CuNCs. Reproduced with permission.^[^ ^95^ ^]^ Copyright 2017, Springer Nature.

For instance, our group for the first time combined the quenching ability of GO to DNA‐templated silver nanocluster (AgNCs) with G4‐enhanced fluorescence enhancing property of NMM together, and fabricated a label‐free and enzyme‐free platform for performing encoder/decoder and a digital comparator, **Figure** **13**A.^[^ ^92^ ^]^ Analogously, Zhang et al. took advantage of above phenomenon, constructed a pG/pC system for intelligent analysis of target miRNA.^[^ ^93^ ^]^ Notably, Liu's group proposed a powerful method in which the AgNCs lighted‐up by poly‐guanine bases and porphyrin dye (NMM) were taken together as a universal system to operate a library of basic logic gates, and they further evolved them into sophisticated circuits to perform advanced arithmetic/non‐arithmetic functions such as HA/HS, multiplexer, and demultiplexer.^[^ ^94^ ^]^ All these works proved the simplicity, high‐efficiency, low‐cost, stable emission, and many other merits of AgNCs to be used as output generator for DNA computing.

In addition to DNA‐AgNCs, poly‐T strands were confirmed as excellent templates to form copper nanoclusters (CuNCs) and have been used as probes of diverse biosensors and DNA logic system. Especially, our group designed a simple, fast, label‐free, and enzyme‐free platform for construction of DNA ternary logic gates with DNA‐CuNCs as output reporter,^[^ ^95^ ^]^ Figure 13B. All DNA inputs are unlabeled single strand without complex design, and the ternary computing can be finished in just 20 min due to the fast formation of CuNCs, which is the highlight of this work. This work largely reduced the time, costs, and complexity during the operation of DNA logic devices.

#### 3.4.5 Other Luminescent Nanomaterials

**Figure 14:**
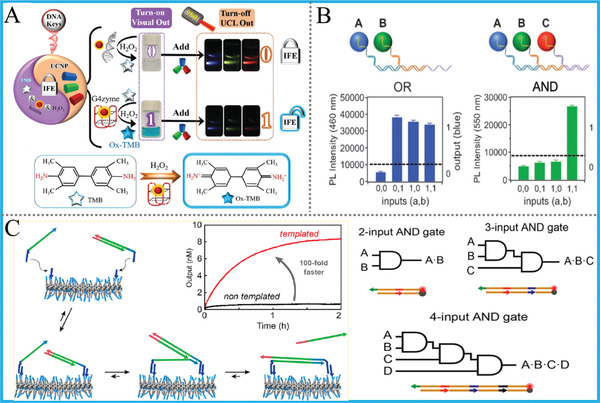
A) Operation of upconversion luminescent DNA logic library. Reproduced with permission.^[^ ^96^ ^]^ Copyright 2019, Royal Society of Chemistry; B) The OR/AND gates based on QDs‐DNA assembly. Reproduced with permission.^[^ ^97^ ^]^ Copyright 2014, Wiley‐VCH; C) Boosting DNA computing by BTA polymer. Reproduced with permission.^[^ ^98^ ^]^ Copyright 2018, American Chemical Society.

Exploring novel illuminators as signal probes of DNA logic computing systems is ever important. Except above nanomaterials, the combination of DNA with other luminescent nanomaterials via covalent/non‐covalent methods also offers novel approaches for DNA computing. Recently, our group reported the first versatile upconversion luminescent (UCL) DNA logic library based on the “DNA‐Unlocked Inner‐Filter‐Effect” (DU‐IFE) between oxidized 3,3’, 5,5’‐tetramethylbenzidine (OxTMB) and UCNPs that matches a “‘lock–key” approach, **Figure** **14**A.^[^ ^96^ ^]^ Remarkably, this library can produce dual‐modal multicolor outputs, wherein RGB colorful UCL ones also can be visualized under NIR‐light, bringing largely enhanced practicability. Through choosing different DNA “keys,” diverse logic devices and series of concomitant arrays are exquisitely achieved. Besides, Ma's group demonstrated the DNA‐programmed dynamic assembly of multicolor quantum dots (QDs) for constructing FRET‐based QDs DNA computing system, in which seven elementary logic gates and a HA derived from their alternative integration were realized, Figure 14B.^[^ ^97^ ^]^ This work offered a typical method to modify DNAs on QDs and further applied the QDs/DNA assembly to DNA computing and other useful applications.

#### 3.4.6 Polymers

Despite the remarkable progress of this area, one of the pending problems is the limited DNA computing speed. Owing to the DNA hybridization and complicated TMSD‐based assembly, it usually takes hours to realize sophisticated logic devices. And this has been one of the non‐negligible drawbacks that hinder the breakthrough of this area.

To address this issue, Merkx's group demonstrated DNA‐functionalized benzene‐1,3,5‐tricarboxamide (BTA) supramolecular polymers as dynamic motifs to boost DNA computing, Figure 14C.^[^ ^98^ ^]^ The kinetics of strand displacement reaction can be accelerated 100‐fold through reasonable design. And the accelerating ability of supramolecular BTA polymers was further illustrated with three famous operations: cascaded AND gates, HCR, and CHA reactions. Similarly, to realize the same goal, Maruyama's group showed that a cationic copolymer, poly(l‐lysine)‐graft‐dextran (PLL‐g‐Dex), can also elevate the speed of DNA logic operations.^[^ ^99^ ^]^ This work reduced the operating time from hours‐level to minutes‐level and also strengthened the nuclease resistance ability of DNA circuits. Although above works evidently improved the computing speed, the used polymers usually need complicated synthesis and purification procedure, this is one problem that needs to be taken seriously in the future.

### 3.5 2D/3D DNA Nanostructures

With the flourishment of DNA nanotechnology that inaugurated by Ned Seeman,^[^ ^100^ ^]^ 2D/3D DNA nanostructures have drawn more and more interests, such as circular/interlocked DNA, DNA tetrahedron/polyhedron, DNA origami and other DNA assemblies.^[^ ^101^ ^]^ Representatively, Damien Woods et al. realized diverse and robust molecular algorithms using reprogrammable DNA self‐assembly in 2019.^[^ ^101^ ^]^ They fabricated a DNA tile set that includes 355 single‐stranded tiles and can be reprogrammed to perform various 6‐bit algorithms. They further fabricated 21 circuits that perform algorithms including copying, sorting, random walking, electing a leader and multifarious innovative logic functions. This work demonstrates that molecular self‐assembly could be a reliable algorithmic element within programmable chemical systems. Considering that Chao's group has made a review on static/dynamic DNA assemblies‐based DNA computing,^[^ ^8^ ^]^ we herein just outline in detail the developments of the first three subjects in this section.

#### 3.5.1 Circular/Interlocked DNA

**Figure 15:**
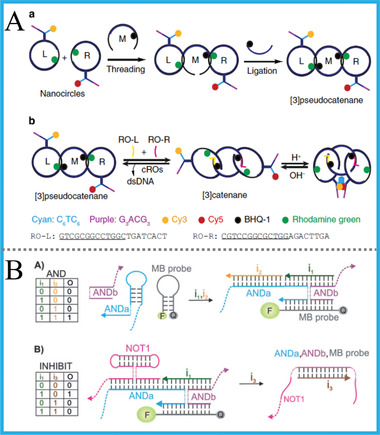
A) Operation of DNA logic gate based on stimuli‐responsive configuration change of circular DNA. Reproduced with permission.^[^ ^103^ ^]^ Copyright 2014, Springer Nature; B) The reusable AND/INHIBIT gates based on tile‐integrated 2D DNA nanostructure. Reproduced with permission.^[^ ^104^ ^]^ Copyright 2016, Wiley‐VCH.

Among multi‐level DNA architectures, circular/interlocked DNAs are one of the most well‐developed 2D DNA structures, and their participation in DNA computing could bring enhanced flexibility, integrability, and universality. For example, Yan's group reported a 3‐input majority gate and multi‐input logic circuits based on a universal triangular DNA (the central calculator) and TMSD reaction.^[^ ^102^ ^]^ Through reasonable design, a complex 5‐input gate and the combination of OR and AND gate was further realized. By utilizing this universal circular DNA as platform, the output signals with better stability and reliability can be obtained. Besides, Li et al. demonstrated the assembly of interlocked DNA nanostructures that controlled by a reversible logic circuit (YES‐AND). By incorporating i‐motif, G4 and exposed toehold into distinct positions of three interlocked DNAs, the configuration of DNA structures can be modulated by pH, complementary DNA and other molecular triggers. **Figure** **15**A.^[^ ^103^ ^]^ Their work not only provided a novel strategy to achieve the controllable assembly of interlocked DNA, but also inspired the construction of DNA logic circuits based on modular DNA structures.

Apart from above circular/interlocked DNAs, Kolpashchikov's group fabricated a tile‐integrated 2D DNA nanostructure for reusable DNA logic computing, in which the DNA gates were operated in a precise order on 2D platform and can work in a reusable format, Figure 15B.^[^ ^104^ ^]^ They envisioned that this work will lay solid foundation for developing DNA nano‐processor, a miniaturized and biocompatible device that could realize complex analysis of different DNA/RNA inputs.

#### 3.5.2 DNA Tetrahedron or Polyhedron

DNA tetrahedron, originated from the assembly of 4 selectively complemented single strands, is one of the simplest 3D DNA nanostructures that created by Turberfield.^[^ ^105^ ^]^ They designed the reconfigurable tetrahedron structures, which inspired subsequent widespread applications of DNA tetrahedron. Owing to its numerous unique merits, such as the rigid configuration, high flexibility and easy synthesis, it has been broadly applied to optical/electrochemical biosensing, intracellular imaging, drug load/release, and so on.

**Figure 16:**
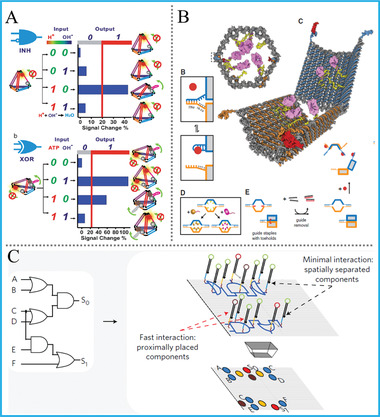
A) The INH and XOR gates based on reconfigurable DNA tetrahedron. Reproduced with permission.^[^ ^106^ ^]^ Copyright 2012, Wiley‐VCH; B) DNA origami‐based AND gate for programmed delivery of loads. Reproduced with permission.^[^ ^108^ ^]^ Copyright, 2012, American Association for the Advancement of Science; C) DNA logic circuit on the basis of spatially localized architecture that operated on DNA origami. Reproduced with permission.^[^ ^109^ ^]^ Copyright, 2017, Springer Nature.

As a well‐known example, Fan's group constructed the first DNA tetrahedron‐based logic platform by combining the reconfigurable design with FRET process and using different triggers (ATP, Hg^2+^, H^+^, OH^−^, and DNAs) as alternative inputs, **Figure** **16**A.^[^ ^106^ ^]^ The AND, OR, INH, XOR gates, and a HA were successfully realized. Due to the necessity of these computing components in biological origin, these reconfigurable logic gates can be further utilized to intracellular sensing and imaging. They achieved the sensitive detection and selective imaging of cellular ATP. This kind of reconfigurable structure brought a vivid model for DNA computing, and also enlightened the reversible spatial control of biological functions.

Apart from DNA tetrahedron, other artificially‐designed 3D DNA nanostructures were also incorporated with DNA computing. For instance, Yao's group designed a 3D DNA nano‐assembly with a triangular prism structure, which can also be considered as a pentahedron.^[^ ^107^ ^]^ By taking the box‐like 3D DNA nano‐prism as a universal platform, various basic logic gates, combinatorial circuits, ternary logic gates and a parity checker for distinguishing even/odd natural numbers were all established. This study not only offered a classical method to make DNA pentahedron, but also a featured model to explore versatile DNA nanodevices based on universal 3D DNA nanostructures.

#### 3.5.3 DNA Origami

DNA origami is a kind of DNA super‐nanostructure that obtained via folding an ultralong single‐stranded “scaffold” into specific shape by hybridizing with hundreds of short “staple” strands. It has been confirmed as an efficient method to achieve 2D/3D DNA nano‐assembly that could enable precise arrangement of different components. As a result of its high integrality, rigid/stable structure, and excellent resistance ability to enzyme digestion, DNA origami has been extensively used as versatile template for molecular robots, nanopattern, intracellular drug delivery, logic computing, and so on.

One famous example was presented by Douglas’ group in 2012 (Figure 16B), they constructed an aptamer‐encoded AND‐gated nanorobot based on DNA origami for targeted delivery of molecular loads.^[^ ^108^ ^]^ The activation of robot was logically controlled by the locks, in which 2 aptamer sequences were encoded to recognize the inputs. Only in the presence of both inputs, the nanorobot can be activated and expose the loads, meeting the feature of an AND gate. And the nanorobot loaded with different antibody components was further applied to 2 types of cell‐signaling stimulation.

Besides, Seelig's group fabricated a spatially localized architecture for fast and modular DNA computing on a DNA origami platform, Figure 16C.^[^ ^109^ ^]^ They operated logic gates and signal transmission channels by spatially locating independent DNA hairpins on a DNA origami. Because of the modularity and universality of this system, various logic gates can be integrated into complex cascaded logic circuits. And the co‐localization of different circuit constituents finally reduced computing time from hours to minutes. This work will inspire the exploration of complicated logic circuits and molecular robots using spatial constraints in the future.

Considering the unique merits of localized DNA circuits, other groups also did excellent works based on DNA origami. For instance, Reif's group presented series of localized DNA hybridization reactions on the surface of a DNA origami rectangle.^[^ ^110^ ^]^ The localization design for their DNA nanodevices does not need DNA strands’ diffusion for each step, resulting in faster reaction kinetics. As localized elements of the devices can be reused in other locations, the locality offered obviously increased scalability. They also envisioned that these localized DNA circuits have the potential to be applied to molecular surfaces (e.g., cells).

## 4 Logic‐Programmed Smart Bio‐Applications

The normal running of miscellaneous biological functions in organism are highly correlated with biochemical reactions and bio‐information processing under specific stimuli. From the perspective of molecular engineers, the above phenomenon belongs to the category of biological logic computing. With the thriving of DNA computing, it has confirmed itself to be more than “game‐playing” attempts and is ready for potential practical applications.^[^ ^7^ ^]^ More and more researchers have turned their attentions to bio‐applications of this biomolecular computer.

### 4.1 DNA Logic‐Based Intelligent Bioanalysis

**Figure 17:**
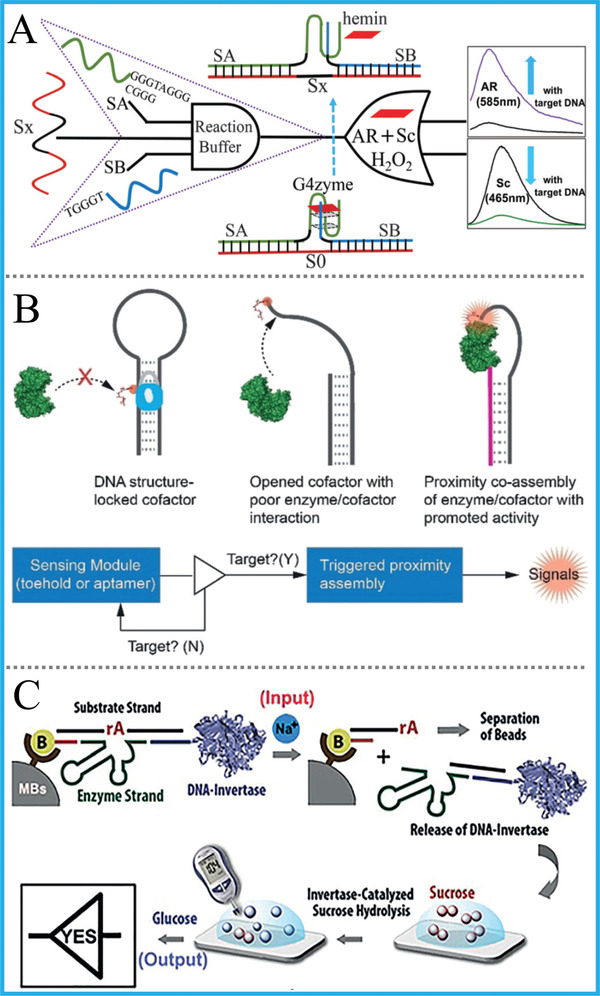
A) Logic‐programmed DNA caliper for ratiometric analysis of DNA. Reproduced with permission.^[^ ^111^ ^]^ Copyright 2016, Royal Society of Chemistry; B) Proximity‐based DNA logic gate for modulating biochemical reactions. Reproduced with permission.^[^ ^113^ ^]^ Copyright 2018, Wiley‐VCH; C) Biocomputing based on DNAzyme and POC devices. Reproduced with permission.^[^ ^114^ ^]^ Copyright 2018, Wiley‐VCH.

For DNA logic‐based intelligent bioanalysis, scientists have witnessed many well‐established paradigms. For example, Tan's group constructed a logic circuit based on aptamer and simple DNA duplex, which can sense local concentration of thrombin and autonomously release a coagulation inhibitor at ultrahigh level of target, thus realizing logic‐guided modulation of thrombin activity.^[^ ^47^ ^]^ This work enlightened the efficient logical modulation of biological functions based on the interaction of aptamer/protein in the future. Notably, our group made use of two fluorescent substrates of G4zyme with inverse responses and fabricated a cascade logic device (a DNA caliper cascades with a 1‐to‐2 decoder) for logic‐programmed ratiometric analysis of target DNA and length measurement, **Figure** **17**A.^[^ ^111^ ^]^ The limit of detection of target DNA is 200 pm, and as low as three bases can be measured under logic control. This work will spur the operation of molecular functional devices for DNA analysis under logic control.

Although above work achieved the logical sensing of specific target DNA, it could not give correct judgement toward complicated situations when many different targets coexist in one sample. To address this issue, Liu's group constructed a reversible Feynman gate by virtue of the combination of AgNCs and G4/NMM.^[^ ^112^ ^]^ Triggered by pathogenic bacterial genes, the logically‐reversible gate was used as an intelligent sensor and the presence of different target genes can be easily distinguished via the one‐to‐one mapping function. Apart from DNAs or genes, DNA‐based logic analysis has also been expanded to other biomolecules. Fu's group fabricated a DNA‐regulated proximity‐based logic circuit for modulation of biochemical reactions, Figure 17B.^[^ ^113^ ^]^ The circuit consisted of a hairpin‐locked catalytic cofactor with prohibited activity. In the presence of specific inputs, the TMSD reaction and aptamer/target interaction will induce a conformational change of DNA lock. The exposed cofactor then could interact with an enzyme and initiate biochemical reaction, enabling the detection of various targets.

In addition to above systems, DNA computing also has been exploited to POC equipment. Representatively, Lu's group transformed biomolecular logic gates into portable, resettable, convenient, and quantitative POC platforms (Figure 17C), in which a personal glucose meter (PGM) worked as signal reporter, the DNAzymes and protein enzymes acted as building components, and glucose/NADH were used as outputs.^[^ ^114^ ^]^ They exhibited the operation of different logic gates and concatenated circuit that respond to various biological molecules (Na^+^, citrate, ATP, and so on). Despite the portable design, the high background signal is a non‐negligible problem that should be further optimized. But this work integrated the inherent merits of both POC device and biomolecular logic, and also pioneered new avenues for smart logical diagnostic devices.

### 4.2 Cell‐Related Bio‐Applications of DNA Logic Computing

With the continuous efforts, the ultimate goal of this field has gradually been disclosed by many experts—“to go to the horizons where silicon could not go” and it is better for scientists to get an in‐depth understanding of organism by introducing DNA computation to cells and tissues.^[^ ^7^ ^]^ Apart from in vitro examples, DNA computing‐based cellular bio‐applications, such as logical imaging, logic‐gated cell‐communication, and cancer therapy, were also extensively explored in recent years.

**Figure 18:**
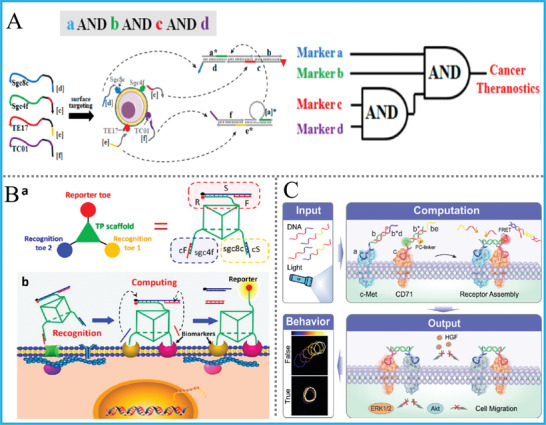
A) DNA logic‐controlled system for cell recognition and targeted therapy. Reproduced with permission.^[^ ^115^ ^]^ Copyright 2015, American Chemical Society.; B) 3D DNA logic nanomachine for computing on target cell surface. Reproduced with permission.^[^ ^116^ ^]^ Copyright 2018, American Chemical Society; C) DNA logic‐directed aptamer‐receptor assembly for modulating cellular signal transduction. Reproduced with permission.^[^ ^118^ ^]^ Copyright 2019, Wiley‐VCH.

Remarkably, Tan's group fabricated a programmable and multiparameter DNA logic system for cancer cell identification and targeted therapy, **Figure** **18**A.^[^ ^115^ ^]^ This DNA device could achieve automatic logical recognition of two/three cell‐surface biomarkers. By integrating DNA aptamers with TMSD reactions, multiple logical analysis of cellular biomarker and logic‐directed photodynamic therapy were realized. Although this strategy provided a facile model for aptamer‐based targeted therapy, the ssDNA and dsDNA are not stable and easy to degrade, causing signal leakage (false‐positive) to some extent. To resolve this problem, in their subsequent work,^[^ ^116^ ^]^ a 3D DNA logical nanorobot was constructed to realize dual‐site recognition and computation on the surface of target cancer cell, Figure 18B. In the presence of specific cell type, the AND gate generates high value, followed by the “*ON*” signal. Otherwise, only “*OFF*” signals will be obtained. In comparison with duplex‐based biomolecular logic circuit, this 3D DNA nanomachine not only avoided the drawbacks of simple DNA structure, but also brought more reliable spatial control, demonstrating better recognition performance and targeting ability.

There are only common logic gates in above systems, whereas complicated logic networks could realize high‐level modulation of biomolecular information on cell membrane. For the cell‐surface‐based applications of DNA computing, Reif's group also fabricated a DNA‐based logic reaction network that works on cancer cell membrane with molecular Boolean response.^[^ ^117^ ^]^ On the basis of their architecture, different kinds of reaction networks were experimentally illustrated, from simple linear cascades to reaction networks of sophisticated structures. They illustrated that these localized DNA reaction networks can be applied to cancer cell detection in a low‐cost, low‐leak, and high signal‐to‐noise ratio manner.

Apart from the specific recognition of aptamer toward target cell and photosensitizer‐induced cell therapy, the bioinformation transmission, and communication between different cells also play crucial roles in organisms. Li's group showed a logic‐controlled aptamer‐receptor assembly for regulating cellular signal transduction, Figure 18C.^[^ ^118^ ^]^ The aptamers were taken as “robotic arms” to interact with target receptors and the DNA logic motif acted as “computer core” to process different inputs. When c‐Met and CD71 were brought into close proximity, the DNA motif will regulate ligand‐receptor interactions of c‐Met and prohibit the functions. Then several logic gates (YES, AND, OR, NOR, two layer AND‐OR circuit) that respond to light or DNA strands were established. This approach offers a novel, non‐genetic and programmable imaging tool to mimic cellular microenvironments for biological and biomedical applications.

Additionally, another work we would like to highlight herein, is the programmed cell adhesion that controlled by on‐chip logic operations reported by Fan's group.^[^ ^119^ ^]^ A kind of cell‐adhesive peptide, named as RGD (Arg‐Gly‐Asp), was modified at the end of single stranded DNAs, which could hybridize with DNA layers that confined on surface. By integrating above strategy with TMSD reaction, they designed in vitro DNA‐based chemical reaction networks (CRNs) to perform logic‐controlled on‐chip cell adhesion. They further fabricated various multi‐input and sequential cellular logic gates. This work provided an efficient technique to synthesize the DNA‐peptide complex, and also offered a universal model for the capture, enrichment, and separation of target cells and regulating biological systems in a logical manner.

Till now, the intelligent in vitro and cellular bio‐applications of DNA computing were vividly demonstrated. Except that, logic‐directed CRISPR gene editing circuits, stimuli‐responsive nanocontainers programmed by logical polymer library and other works were also reported^[^ ^120^, ^121^ ^]^ These works integrated both logic's stringency and excellent biocompatibility of biomaterials, and will lay robust foundation for logic‐guided other applications in the future.

## 5 Conclusions and Future Perspective

In this review, we attempted to summarize recent developments in DNA computing. We first illustrated the operating principles of different DNA logic devices, then by taking miscellaneous “building‐block” materials as classification standard, we systematically summarized and reviewed state‐of‐the‐art advancements of innovative DNA computing systems and their smart bio‐applications, in which the famous or representative examples of each category were alternatively presented and discussed.

Despite the burgeoning achievements in this area, there still exist non‐negligible “Achilles’ heels.” From our point of view, current bottlenecks and potential perspectives in this area are illustrated below. 1) During most DNA logic systems, the probe‐labeled strands, natural enzymes or tool‐enzymes are frequently used, resulting in high‐cost and ineluctable time‐wastage. Thus, exploring label‐free, enzyme‐free, and cost‐effective logic platform remains further efforts. 2) Although the widely‐used TMSD reaction shows acceptable efficiency, it usually needs unique and complex sequence design, long reaction time, and also exhibits signal‐leakage to some degree. Therefore, searching for novel DNA hybridization strategy or optimized logical systems with high signal‐to‐noise ratio that could eliminate above drawbacks is the key direction. 3) Typically, previous logic platforms can only perform limited logic devices, designing versatile systems for executing multiplex (or pluralistic) logic functions is another promising approach to address this issue. 4) Majority of DNA logic systems are still confined to proof‐of‐principle or test‐tube stage and could not compete with already mature silicon circuits. Exploiting DNA computation to cellular applications or tissue engineering for “real‐smart” diagnostics/therapy has been confirmed as one of the most meaningful goals, but needs elegant design and great endeavors.

We admit that this prosperous area is still in its infancy and there are many remaining limitations to be conquered. But we believe that, under the propelling of multifarious advanced functional materials, DNA computing will not only exhibit surprising magic power in specific areas and intelligent applications, but also definitely reach a new‐level in the not‐too‐distant future. We hope this review has given a comprehensive understanding of logic principles, latest progress and bio‐applications of DNA computing, opening up inspiring horizons for researchers in this area to engineer the next‐generation molecular/DNA computers.

## Conflict of Interest

The authors declare no conflict of interest.
